# Free Amino Acid and Volatile Compound Profiles of *Jeotgal* Alternatives and Its Application to Kimchi

**DOI:** 10.3390/foods10020423

**Published:** 2021-02-15

**Authors:** Hye Jin Lee, Min Jung Lee, Yun-Jeong Choi, Sung Jin Park, Mi-Ai Lee, Sung Gi Min, Sung-Hee Park, Hye-Young Seo, Ye-Rang Yun

**Affiliations:** 1 Industrial Technology Research Group, World Institute of Kimchi, Gwangju 61755, Korea; hjl0617@wikim.re.kr (H.J.L.); leemj@wikim.re.kr (M.J.L.); yjchoi85@wikim.re.kr (Y.-J.C.); parksungjin@wikim.re.kr (S.J.P.); leemae@wikim.re.kr (M.-A.L.); skmin@wikim.re.kr (S.G.M.); shpark@wikim.re.kr (S.-H.P.); 2 Hygienic Safety and Analysis Center, World Institute of Kimchi, Gwangju 61755, Korea; hyseo@wikim.re.kr

**Keywords:** *Jeotgal* alternative, kimchi, glutamic acid, volatile compound, savory taste

## Abstract

*Jeotgal* containing abundant free amino acids plays an important role in the unique savory taste (umami) and flavor in kimchi. However, it is also responsible for the unpleasant fishy smell and high salt content of kimchi. Therefore, the present study aimed to identify alternative *jeotgal* sources and investigate the fermentation properties of *jeotgal* alternatives added to kimchi. The tomato hot-water extract (TH2) and dry-aged beef hot-water extract (DBH) were selected as *jeotgal* alternatives for kimchi preparation based on their glutamic acid contents. Characteristics of kimchi with TH2 alone (JA1) and TH2 and DBH in combination (1:1, JA2) were compared with kimchi prepared using commercially available anchovy fish sauce (CON). The pH of JA1 and JA2 was slightly decreased during fermentation, whereas the salinity was significantly lower than CON (*p* < 0.05). Notably, the most effective factor of the savory taste of kimchi, glutamic acid contents of JA1 and JA2 were significantly higher than that of CON (*p* < 0.05). In conclusion, JA1 showed slower fermentation with lower salinity and higher glutamic acid content than CON. Overall, this study showed that JA1 derived from TH2 could improve the taste and quality of kimchi by increasing glutamic acid content and decreasing the unpleasant flavor.

## 1. Introduction

*Jeotgal* (salted seafood) is a fermented food obtained by degradation of protein peptides by its enzymes and external microorganisms, imparting a unique flavor and taste to kimchi, while spoilage is prevented by adding large amounts of salt [1]. Thus, *jeotgal* is an important ingredient in kimchi. It contains a large amount of glutamic acid and nucleic acid-related substances such as adenosine triphosphate (ATP), adenosine diphosphate (ADP), and inosine monophosphate (IMP), responsible for the savory taste of kimchi [2,3,4,5]. However, the high salinity and unpleasant fishy smell of *jeotgal* makes it difficult to prepare standardized kimchi and reduces customer preferences [6], warranting the need for alternative ingredients to prepare *jeotgal*. Therefore, it has recently gained increasing interest. Several studies focused on addressing these problems associated with the high salinity and fishy smell while searching for alternative *jeotgal* that could impart on kimchi a taste similar to that of original *jeotgal* [7]. However, most of these studies on alternative ingredients for *jeotgal* have mainly demonstrated the potential of seafood and fishery products. On the contrary, the use of vegetable protein sources and/or animal protein sources as alternative *jeotgal* sources has not been explored yet, although studies have identified them as potential sources of umami compounds [8,9].

Apart from the previous studies, food ingredients other than seafood and fishery products containing high glutamic acid, which is closely related to savory taste, have been widely studied. Skurray et al. [10] studied the glutamic acid content in fresh and processed foods and reported that the glutamic acid of ripe tomato increased about 6-fold compared to that of fresh tomato. Al-Sayed et al. [11] also reported that ripe tomato is the richest source of glutamic acid among fruits and contains approximately 300 mg of glutamic acid per 100 g of ripe tomatoes. Therefore, tomato is highly utilized to produce the rich flavor of broth overseas with plenty of glutamic acids, organic acid, and other ingredients [12]. As another alternative ingredient, beef is generally known to improve the savory taste according to the aging method. Dry-aging is a process where beef is placed with good air circulation at 2–4 °C and 65–85% humidity for 20–60 days. The weight is decreased, however, the quality of beef is softened and the savory taste increases [13]. It has been shown that the free amino acid content of beef after drying and vacuum-packing increased about four times along with an increase in functional peptides such as taurine, anserine, and carnosine. In particular, the glutamic acid and IMP related to the savory taste were the highest at 40 days into the dry-aging period [14]. 

Here, we aimed to make kimchi using *jeotgal* alternatives to improve the taste and reduce the fishy smell. In this study, beef, soybean, and tomato were selected as alternative ingredients for *jeotgal* preparation. Protein hydrolysis processes such as enzymatic hydrolysis and hot water extraction were initially conducted to improve the savory taste. The final *jeotgal* alternatives for kimchi application were selected based on their glutamic acid contents. Then, physicochemical, microbial, free amino acid, and volatile compounds of kimchi prepared with *jeotgal* alternatives were investigated and compared to those of kimchi prepared with commercial *jeotgal*.

## 2. Materials and Methods

### 2.1. Materials and Reagents

The *jeotgal* alternative materials—beef, soybean, and tomato—were purchased from an on-line market (G-market, Seoul, Korea). Proteases (Protamex, Flavourzyme, and Alcalase) were purchased from Novo Nordisk (Bagsvaerd, Denmark). A glutamic acid assay kit was purchased from Megazyme (K-GLUT, Ireland). The kimchi ingredients, including brined kimchi cabbage, red pepper, garlic, ginger, and anchovy fish sauce, were purchased from Seobu Agricultural and Fishery Market in Gwangju (Gwangju, Korea). MRS and PCA agar plates were purchased from Kisan Bio (Seoul, Korea).

### 2.2. Preparation of Jeotgal Alternative

The *jeotgal* alternatives were prepared using dry-aged beef (DB), isolated soybean protein (ISP), and tomatoes in optimized powder form obtained by freeze-dying. To improve the savory taste, enzymatic hydrolysis and hot-water extraction were used as a protein processing method in each *jeotgal* alternative source. DB mixed with 2-folds distilled water was extracted with hot water at 90 °C for 30 min. The solution was then filtered and centrifuged at 4000 rpm for 30 min. Then the supernatant was collected and freeze-dried, and the preparation was designated as DB hot water extract (DBH). Subsequently, the pellet obtained after separating the supernatant was hydrolyzed using three different protease enzymes—flavourzyme, protamex, and alcalase—each at a concentration of 1% was used for the enzymatic hydrolysis at 50 °C for 2 h. The hydrolyzed products were freeze-dried, and the preparations were named DBHF, DBHP, and DBHA, respectively. ISP with 10-folds distilled water were hydrolyzed using the three proteases (flavourzyme, protamex, and alcalase; 1% each) at 50 °C for 4 h, inactivated at 95 °C for 30 min, and centrifuged at 4000 rpm for 30 min. The supernatant was then collected and freeze-dried, and the preparations were named ISPF, ISPP, and ISPA, respectively. The mature tomatoes with 2-folds distilled water were extracted with hot-water at 90 °C for 1, 2, 3, or 4 h, filtered, and centrifuged at 4000 rpm for 30 min. The supernatant was then collected and freeze-dried, and the preparations were named TH1, TH2, TH3, and TH4, respectively.

### 2.3. Glutamic Acid Content Analysis

To select the final *jeotgal* alternative, glutamic acid was measured using a Glutamic acid assay kit after protein processing. Each of the *jeotgal* alternative (10 µL) and standard (10 µL) was initially reacted with buffer (50 µL), nicotinamide-adenine dinucleotide/iodonitrotetrazolium chloride (NAD + /INT, 20 µL), diaphorase (5 µL), and distilled water (200 µL) for 2 min. Absorbance (A1) was read at 492 nm using a SPECTROStar Nano microplate reader (BMG LABTECH, Ortenberg, Germany). After reacting with glutamate dehydrogenase (GIDH, 5 µL), absorbance (A2) was read at 492 nm. Glutamic acid content was calculated using the following formula:

Glutamic acid content = [(A2−A1) of sample/(A2−A1) of standard × standard]

Subsequently, the *jeotgal* alternative with a high glutamic acid content in each of the alternative materials was selected and their free amino acid and volatile compounds were measured compared with those of anchovy fish sauce (CON).

### 2.4. Free Amino Acids Analysis

For free amino acid analysis, the *Jeotgal* alternatives and kimchi samples were diluted, homogenized, and refrigerated for 15 h. The solutions were then filtered using 0.45 µm membrane filters, and the filtrate was used as the samples for further analysis. Free amino acids were analyzed using high-pressure liquid chromatography (HPLC, Ultimate 3000, Thermo Dionex, USA) equipped with a VDSpher 100 C18-E column (4.6 × 150 mm, 3.5um/VDS optilab, Germany), at an oven temperature of 40 °C. The operational conditions for HPLC are shown in (Table 1). The amino acids were quantified by calibrating with standard amino acids (Agilent Technologies, CA, USA).

### 2.5. Volatile Compounds Analysis

Volatile compounds extracted using solid-phase microextraction (SPME) fibers (DVB/CAR/PDMS, 50/30 µM, Supelco-57329-U) were analyzed using a gas chromatography-mass spectrometer (GC/MS 7890A, Agilent Technologies, SC, USA) with an autosampler (Multi-Purpose Sample with DHS option, MPS, Gerstel, Germany). All samples were placed in a 10 mL, sealed with an aluminum cap, and then absorbed onto polydimethylsiloxane (PDMS) fiber at 100 rpm for 30 min at 50 °C. The SPME fiber, which extracted the volatile compounds, was automatically injected into a gas chromatography-mass spectrometer (GC/MS) injection port, and the volatile compounds were thermally desorbed at 250 °C for 3 min and analyzed using GC/MS. Electron impact ionization (70 eV) was performed at a full scan range of 50–550 *m*/*z*. The volatile compound was identified based on a mass spectral library (WILEY 10N). The operational conditions for GS/MS analysis are shown in (Table 2).

### 2.6. Preparation of Kimchi by Adding Jeotgal Alternative

Based on the high glutamic acid content, DBH and TH2 were selected for kimchi preparation (see Section 3.1). According to the kimchi recipe (Table 3), kimchi was prepared by replacing anchovy fish sauce with TH2 alone (JA1) or TH2 and DBH (1:1; JA2), and kimchi with anchovy fish sauce was used as a control (CON). Kimchi was stored and fermented at 10 °C for 2 weeks, and its physicochemical properties, microbial properties, free amino acid contents, and volatile compound composition were examined on weeks 0, 1, and 2, where week “0” represents the data before fermentation.

### 2.7. Physicochemical Analysis

The pH was measured with a pH meter (pH Electrode blue line 12, SCHORR Instrument, Germany) after kimchi sample blending, and total acidity was titrated with 0.1 N NaOH until pH reached 8.3. Salinity was measured by Mohr’s titration [15]. In brief, the kimchi juice was diluted with distilled water (1/100) and then filtered. After adding 2% potassium chromate as the indicator (1 mL), the mixture was titrated against 0.02 N AgNO_3_ until reaching the endpoint based on a detectable change to a red-brown color.

### 2.8. Microbial Analysis

Microbial analysis for total viable bacteria and lactic acid bacteria was conducted using a pre-made plate count agar (PCA) plate and deMan-Rogosa Sharpe (MRS) agar plate. To measure the total viable bacteria, kimchi samples were diluted to 10^1^ to 10^7^. Diluted kimchi samples (100 μL) were evenly spread on a PCA plate (Kisan Bio, Seoul, Korea) and incubated at 37 °C for 2 days. Total viable bacteria was measured by counting in the cultured PCA plate. To measure the lactic acid bacteria, diluted kimchi samples were inoculated on an MRS agar plate (Kisan Bio, Seoul, Korea), and then incubated at 37 °C for 2 days under an anaerobic condition. After incubation, the lactic acid bacteria in the MRS agar plate was counted.

### 2.9. Statistical Analysis

Each experiment was conducted in triplicate, and the results were expressed as the mean and standard deviation (SD). All data were analyzed one-way analysis of variance analysis (ANOVA), and significant differences were analyzed by Duncan’s multiple range test (*p* < 0.05) using the GraphPad Prism 7 (GraphPad Software Inc., San Diego, CA, USA).

## 3. Results and Discussion

### 3.1. Glutamic Acid Content of Jeotgal Alternative

**Figure 1:**
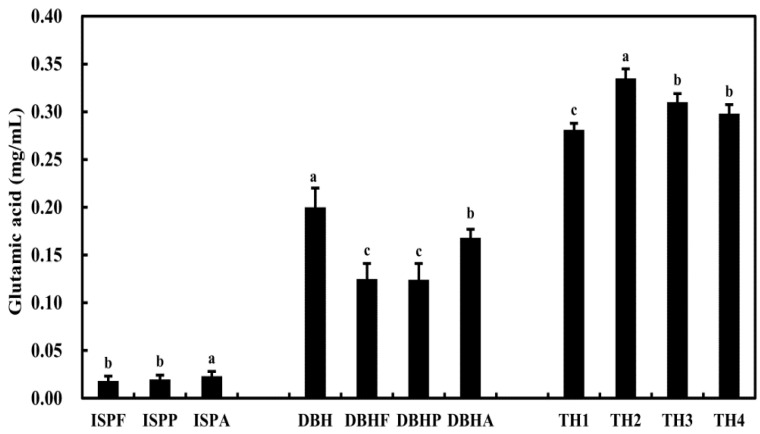
Glutamic acid content of *jeotgal* alternative materials after the enzymatic digestion process. Isolated soybean protein (ISP), dry-aged beef (DB), and tomato (T). Data are expressed as means ± SD. Different lowercase letters indicate a significant difference in the same *jeotgal* alternative (*p* < 0.05).

The glutamic acid contents of the *jeotgal* alternatives are shown in (Figure 1). Among the enzymatically digested ISP, ISPA showed the highest glutamic acid as 0.02 mg/mL, consistent with Valerio’s findings [16], which showed a similar low glutamic acid content in the formulation of yeast-leavened bread. Hot water extraction in DB significantly increased glutamic acid content compared with enzymatic digestion (*p* < 0.05), however, among the enzymatically hydrolyzed preparations, DBHA showed the highest glutamic acid content. In the TH sample extracted with hot water (TH1-TH4), glutamic acid content was significantly increased in TH2 compared to the other three formulations (*p* < 0.05). Overall, among the *jeotgal* alternatives, the glutamic acid content of ISPA was relatively lower than that of other alternatives. Taken together, the findings revealed the highest glutamic acid content in ISPA, the preparation obtained by enzymatic hydrolysis of ISP with 1% alcalase at 50 °C for 4 h. Of the hot water extract and enzymatically hydrolyzed preparations of DB, DBH had a higher glutamic acid content than DBHA, DBHP, and DBHF, wherein the glutamic acid content of DBHA was maximum among the three enzymatically hydrolyzed preparations of DB. Moreover, of the four ripe tomato hot-water extracts, TH2 showed the highest glutamic acid content. Based on these results, ISPA, DBH, and TH2 were initially selected as *jeotgal* alternatives for the kimchi preparation.

ISPF, ISPP, and ISPA, Enzymatic hydrolysis of ISP with flavourzyme, protamex, and alcalase at 50 °C for 4 h. DBH, DB with hot water extraction at 90 °C for 2 h; DBHF, DBHP, DBHA, enzymatic hydrolysis of DBH with flavourzyme, protamex, and alcalase at 50 °C for 2 h. TH1, TH2, TH3, and TH4, tomato with hot water extraction at 90 °C for 1, 2, 3, and 4 h.

### 3.2. Free Amino Acid Composition of Selected Jeotgal Alternative

The amino acids, including glutamic acid, alanine, and glycine, are the main components that contribute to the unique savory taste of *jeotgal,* whereas the amino acids, including leucine, isoleucine, and arginine, responsible for bitterness, are known to affect the quality of *jeotgal* negatively [1]. As shown in (Table 4), TH2 showed the highest glutamic acid contents at 33328.32 mg/kg, followed by commercial *jeotgal* at 21032.41 mg/kg. The glycine content was the highest in DBH (4025.58 mg/kg) among the *jeotgal* alternatives; however, it was similar to that of commercial *jeotgal* (4841.74 mg/kg). The alanine content of DBH (14997.51 mg/kg) was higher than that of commercial *jeotgal* (9501.16 mg/kg). However, the contents of these three amino acids were lowest in ISPA. Furthermore, the contents of leucine, isoleucine, and arginine were the highest in commercial *jeotgal*, followed by DBH, TH2, and ISPA, in that order. The content of most of the amino acids was low in ISPA in addition to the maximum number of not detectable (ND) amino acids that included taurine and gamma-aminobutyric acid (GABA). As shown in (Table 4), taurine and GABA were detected only in CON and TH2, respectively. Taken together, TH2 or DBH alone or in combination were speculated to maintain the savory taste of commercial *jeotgal* and reduce the bitterness when used in kimchi. Therefore, TH2 and DBH were used as high potential *jeotgal* alternatives for kimchi preparation. 

### 3.3. Volatile Compounds Composition of Jeotgal Alternatives

Volatile compounds of *jeotgal* alternatives were identified and quantified using the internal standard materials. As shown in Table 5, volatile compounds in CON were detected in the order of 3-methylbutanoic acid, 2,4-bis(1,1-dimethylethyl) phenol, and butanoic acid. Among these, 3-methylbutanoic acid is closely related to the unpleasant flavor of *jeotgal* and imparts a rancid, cheesy, sweaty flavor [17]. In a dry porcini mushroom (*Boletus edulis*) study, 3-methylbutanoic acid has also been described to impart a lactic or sweaty aroma [18], whereas butanoic acid provides a cheesy note to roasted shrimp [19]. Furthermore, the volatile compound composition of DBH identified 2,4-bis(1,1-dimethylethyl) phenol as the major compound, followed by trimethylpyrazine and butanoic acid. Of these, trimethylpyrazine has been identified as a flavor compound in roasted coffee beans, cooked rice, and soy paste [20,21]. In ISPA, only 2,4-bis(1,1-dimethylethyl) phenol and acetic acid were detected; however, the other volatile compounds were hardly detected. The major volatile compound of TH2 was 2,4-bis(1,1-dimethylethyl) phenol, followed by acetic acid at a low content. These results demonstrated a more or less similar profile of volatile compounds in CON and DBH, which have animal protein bases, which differed significantly from that of ISPA and TH2, having vegetable protein bases. It is noteworthy that 3-methylbutanoic acid was dramatically decreased in all three *jeotgal* alternatives, indicating their potential to reduce the unpleasant fishy smell of kimchi. However, 2,4-Bis(1,1-dimethylethyl) phenol and butanoic acid are significantly higher in DBH than those in TH2. Taken together, it can be inferred that TH2 with a desirable profile of volatile compounds and higher glutamic acid content than commercial *jeotgal* could be the best *jeotgal* alternative.

### 3.4. pH, Total Acidity, and Salinity of Kimchi Prepared with Jeotgal Alternatives

Table 6 shows the pH, total acidity, and salinity of kimchi prepared with the *jeotgal* alternatives—JA1 and JA2. On week 0, the pH of JA2 was significantly higher than that of CON and JA1. However, the pH of JA1 was the lowest (4.54), and this could be due to the influence of the ingredients of JA2, such as tomatoes, which are acidic foods [22]. On week 1, the pH of CON was rapidly decreased to 4.35, while that of JA1 was decreased to 4.41. The pH of JA2 was also decreased, but the decrease was less than that of the CON. Similarly, on week 2, the pH of the CON decreased rapidly compared to JA1 and JA2. These results indicated the effect of *jeotgal* alternatives on fermentation delay by slowing the decrease in pH. In general, the pH was changed due to the organic acid produced by the decomposition of carbohydrates in kimchi ingredients and affected the taste of kimchi. These results are consistent with a previous study that reported similar trends of decrease with the progression of the kimchi fermentation process [23].

Total acidity was gradually increased in all groups, and JA2 showed the highest total acidity of 1.36% on week 2. Surprisingly, the salinity showed a significant difference among groups. Compared to CON (1.87%), the salinities of JA1 and JA2 were significantly lower (*p* < 0.05, 1.13%, and 1.03%, respectively). These results indicate that *jeotgal* alternatives can improve the high salinity problem of *jeotgal* by reducing the salinity of kimchi. Taken together, the findings suggest that the *jeotgal* alternatives could lower the saltiness of kimchi and delay its fermentation.

### 3.5. Microbial Profiles of Kimchi Prepared with Jeotgal Alternatives

The microbial changes are shown in (Table 7). The total viable cell showed a similar level in different groups (6.93~7.02 CFU/mL) on week 0, which was increased on week 1 in all groups; however, CON showed the highest level (8.19 CFU/mL). On week 2, the total viable cell increased from 8.49 to 8.83 CFU/mL and was the highest in JA1. Similarly, lactic acid bacteria showed similar results. CON and JA1 showed the highest lactic acid bacteria on weeks 1 and 2, respectively. Inconsistent with these results, Choi et al. reported that total viable cell counts reached the maximum of approximately 15 to 18 days and then rapidly decreased [24]. In the present study, the microbial changes were similar between CON and *jeotgal* alternatives at 10 °C, and the lactic acid bacteria of *jeotgal* alternative was higher than CON on week 2. These results indicated that *jeotgal* alternatives could maintain kimchi fermentation qualities by preserving the microbial properties.

### 3.6. Free Amino Acid Composition of Kimchi Added Jeotgal Alternatives

Changes in free amino acids were summarized in (Table 8). On week 0, the glutamic acid content of JA1 was the highest at 1246.23 mg/kg. Even though kimchi was prepared with a similar amino acid level in all groups, the glutamic acid content of JA1 and JA2 was higher than that of CON on week 0. In particular, the glutamic acid content of JA1 was the highest during the kimchi fermentation period, which was approximately 3-folds higher than that of CON. Regarding glycine content, CON showed the highest level on week 0, wherein JA2 showed the highest level on weeks 1 and 2. Similarly, JA2 showed the highest alanine content on all weeks. Consistent with the result of week 0, free amino acid contents of JA1 and JA2 were higher at 1 and 2 weeks than in CON. Notably, leucine and isoleucine contents, contributing to bitterness [4], were lower in JA1 and JA2 than CON on week 1; however, the arginine content was slightly higher than that in CON. Similar results were also observed on weeks 0 and 2. According to a previous study, these changes in free amino acid during kimchi fermentation are mainly caused by a proteolytic reaction of the protein of *jeotgal* and greatly affect the taste of kimchi [25,26]. Uniquely, JA2 showed the highest taurine content in all weeks. Moreover, the GABA content in JA1 and JA2 was higher than that in CON. Based on these results, it can be inferred the *jeotgal* alternatives could increase the savory flavor of kimchi, leading to improved kimchi quality.

### 3.7. Volatile Compounds Composition of Prepared with Jeotgal Alternatives

The composition of the volatile compounds is shown in (Table 9). A total of 56 volatile compounds were detected in kimchi added *jeotgal* alternatives for 2 weeks. On week 0, 4-isothiocyanato-1-butene showed the highest content in all groups, followed by 2-isothiocyanato-ethylbenzene. Both 4-isothiocyanato-1-butene and 2-isothiocyanato-ethylbenzene dramatically decreased during kimchi fermentation in all groups. 4-isothiocyanato-1-butene is mainly produced by sinigrin hydrolysis of glucosinolates during the crushing and cooking process of cabbage and exhibits the characteristic and irritating flavor of the cruciferous plant [27]. Moreover, 3,7-dimethyl-2,6-octadienal, methyl 2-propenyl trisulfide, and zingiberene were decreased, while 2,3-butanediol, diallyl disulfide, and acetic acid were increased during kimchi fermentation. Interestingly, methyl 2-propenyl trisulfide showed the highest content on week 1 and then decreased on week 2. Consistent with our results, several previous studies have reported high contents of sulfur compounds in kimchi [28,29,30,31]. For example, Kang et al. reported high contents of sulfur compounds such as allyl methyl sulfide, dimethyl disulfide, methyl propyl disulfide, dimethyl trisulfide, and di-2-propenyl trisulfide in kimchi [28]. 

Furthermore, the contents of most of the volatile compounds in JA1 added kimchi were less than those in CON added kimchi. Methyl 2-propenyl trisulfide, a unique volatile component of raw garlic [32], was reduced by adding *jeotgal* alternatives on all weeks. Moreover, 3,7-dimethyl-2,6-octadienal, shown to impart a strong lemon flavor [33], was reduced with the addition of JA1 by approximately 30% compared to CON and rapidly decreased during kimchi fermentation. Unexpectedly, 4-isothiocyanato-1-butene content of kimchi with JA1 increased by 18% on weeks 0 and 1 compared to CON; however, it was hardly detected on week 2. Though acetic acid and bezenepropanentrile contents were increased on weeks 1 and 2, they were lower than those in CON. The levels of most volatile compounds in JA2 added kimchi were similar to those in the CON, including 2-isothiocyanato-ethylbenzene, 4-isothiocyanato-1-butene, and methyl 2-propenyl trisulfide. Among volatile compounds, 4-Isothiocyanato-1-butene, (2-isothiocyanatoethyl)benzene, and 3,7-Dimethyl-2,6-octadien-1-ol of JA1 are significantly decreased on week 0. 4-isothiocyanato-1-butene is described as a gasoline-like flavor and isothiocynates are mainly described as the strong mustard-like pungent flavor. Hence, a significant reduction would be a positive effect of JA1. Overall, the findings revealed suggesting JA1 as a potential *jeotgal* alternative for kimchi application.

## 4. Conclusions

In this study, soybean protein, beef, and tomato were used as potential sources for *jeotgal* alternatives. Initially, they were either extracted through hot water or enzymatically hydrolyzed using three different proteolytic enzymes (Alcalase, Protamex, and Flavourzyme). The findings revealed that glutamic acid content, the umami amino acid was higher in DBH, ISPA, and TH2 than the other preparation obtained from the same source. Profiling of the free amino acids and the volatile compounds in the above three preparation identified DBH and TH2 as potential alternatives to replace CON. Subsequently, kimchi was prepared by adding either TH2 alone (JA1) or in combination with DBH (JA2). The addition of JA1 and JA2 showed a slow decrease in pH and low salinity compared to CON. In addition, JA1 and JA2 added kimchi showed higher glutamic acid contents than CON added kimchi, indicating the effects of JA1 and JA2 on the improved savory taste of kimchi. Futrthermore, JA1 and JA2 showed a similar or improved composition of volatile compounds compared with CON. Collectively, these findings suggest that JA1 containing TH2, a *jeotgal* alternative, could improve kimchi flavor and quality by reducing off-flavor and salinity.

## Figures and Tables

**Table 1:** Operational conditions for high-pressure liquid chromatography (HPLC) analysis to quantify free amino acid contents.

Parameters	Conditions
HPLC system	Ultimate 3000
Column	VDSpher 100 C18-E
Mobile phase	A: 40 mM sodium phosphate dibasic, pH 7.8
	B: water/acetonitrile/methanol (10:45:45 *v*/*v*%)
Flow rate	1.5 mL/min
Detector	FL Detector 1260 FLD
	: Emission 450 nm, Excitation 340 nm (OPA)
	: Emission 305 nm, Excitation 266 nm (FMOC)
	UV Detector
	: 338 nm
Oven Temperature 40 °C	

**Table 2:** Operational conditions for gas chromatography-mass spectrometer (GS/MS) analysis to quantify volatile compounds.

Parameters	Conditions
GC System with MS	Agilent 7890 with 5977BA
Separation column	DB-WAX (60 m × 0.25 mm × 0.25 µM)
Injection temperature	250 °C
Carrier gas flow	1 mL/min (He)
Oven temperature	40 °C (3 min) → 150 °C (2 °C /min)(10 min) → 200 °C (4 °C/min)(10)

**Table 3:** Ingredients used to prepare kimchi with commercial and *jeotgal* alternatives.

Ingredient	CON (g)	JA1 (g)	JA2 (g)
Brined Kimchi cabbage	700	700	700
Red pepper	66	66	66
Garlic	36	36	36
Ginger	10.2	10.2	10.2
Rice paste	57.3	57.3	57.3
Water	98.1	98.1	98.1
Sugar	1.5	1.5	1.5
Salt	2.28	2.28	2.28
*Jeotgal*	28.62	-	-
DBH	-	-	14.31
TH2	-	28.62	14.31
Total	1000	1000	1000

**Table 4:** Free amino acid composition of *jeotgal* alternatives.

Free Amino Acids	CON (mg/kg)	DBH (mg/kg)	ISPA (mg/kg)	TH2 (mg/kg)
Aspartic acid	12,591.76 ± 65.40 ^a^	488.85 ± 31.64 ^c^	57.78 ± 3.00 ^d^	8425.89 ± 77.47 ^b^
Glutamic acid	21,032.41 ± 407.62 ^b^	7815.70 ± 332.44 ^c^	192.49 ± 9.41 ^d^	33,328.32 ± 212.79 ^a^
Asparagine	85.72 ± 1.90 ^c^	2152.63 ± 110.63 ^b^	110.74 ± 4.28 ^c^	4515.82 ± 21.08 ^a^
Serine	6548.43 ± 78.05 ^a^	5163.78 ± 306.21 ^a^	132.22 ± 6.92 ^c^	1169.69 ± 6.54 ^b^
Glutamine	117.89 ± 4.17 ^c^	18,764.66 ± 945.22 ^a^	37.89 ± 0.46 ^d^	6278.53 ± 8.15 ^b^
Histidine	6141.59 ± 250.82 ^a^	2128.26 ± 105.86 ^b^	122.26 ± 1.35 ^d^	773.99 ± 0.41 ^c^
Glycine	4841.74 ± 8.73 ^a^	4025.58 ± 464.57 ^a^	26.02 ± 1.87 ^c^	160.82 ± 3.76 ^b^
Threonine	7394.65 ± 48.64 ^a^	3983.79 ± 173.73 ^b^	31.62 ± 0.62 ^d^	1997.02 ± 3.13 ^c^
Arginine	6246.24 ± 22.23 ^a^	4173.57 ± 655.57 ^b^	212.44 ± 18.74 ^d^	1468.70 ± 43.62 ^c^
Alanine	9501.16 ± 35.15 ^b^	14,997.51 ± 655.57 ^a^	145.19 ± 5.38 ^d^	666.53 ± 35.05 ^c^
Taurine	2189.53 ± 38.50	ND	ND	ND
GABA	ND	ND	ND	7467.79 ± 139.30
Tyrosine	668.90 ± 64.66 ^b^	2976.79 ± 86.99 ^a^	126.07 ± 28.41 ^d^	474.99 ± 37.33 ^c^
Valine	7689.68 ± 26.09	4440.78 ± 121.56	ND	324.92 ± 9.16
Methionine	2744.33 ± 2.94	2543.58 ± 100.97	ND	193.65 ± 9.11
Tryptophane	1721.02 ± 13.16	845.61 ± 53.55	ND	469.91 ± 10.98
Phenylalanine	4437.97 ± 40.53	3226.94 ± 158.98	ND	1650.90 ± 22.61
Isoleucine	4919.20 ± 52.41	3221.46 ± 124.82	ND	563.90 ± 4.58
Leucine	6295.06 ± 47.16 ^a^	5884.54 ± 249.84 ^a^	128.94 ± 1.70 ^c^	782.21 ± 10.40 ^b^
Lysine	11,471.60 ± 40.38 ^a^	6181.05 ± 1019.20 ^b^	221.65 ± 7.68 ^d^	782.21 ± 10.40 ^c^
Proline	2565.24 ± 122.12	2629.67 ± 380.10	ND	192.56 ± 12.43

ND: not detected. Data are expressed as mean ± SD. Different letters are significantly different among groups (*p* < 0.05).

**Table 5:** Volatile compounds composition of commercial *jeotgal* (CON) and *jeotgal* alternatives (DBH, ISPA, and TH2).

Volatile Compounds ^1^	CON (Peak Area)	DBH (Peak Area)	ISPA (Peak Area)	TH2 (Peak Area)
2,4-Bis(1,1-dimethylethyl) phenol	0.53 ± 0.19 ^NS^	2.68 ± 0.75	0.07 ± 0.00	0.46 ± 0.15
2,5-Dimethylpyrazine	0.12 ± 0.04 ^b^	0.30 ± 0.10 ^a^	0.00 ± 0.00 ^c^	0.00 ± 0.00 ^c^
2-Furanmethanol	0.14 ± 0.05	ND	ND	0.01 ± 0.00
2-Methylpentanoic acid	0.11 ± 0.04	ND	0.00 ± 0.00	ND
2-Methylpropanoic acid	0.14 ± 0.05	0.03 ± 0.01	ND	ND
3-Methylbutanoic acid	1.79 ± 0.57 ^a^	0.06 ± 0.02 ^b^	0.00 ± 0.00 ^b^	0.01 ± 0.00 ^b^
4-Methylpentanoic acid	0.10 ± 0.03	0.01 ± 0.00	ND	ND
Acetic acid	0.11 ± 0.04 ^b^	0.42 ± 0.14 ^a^	0.01 ± 0.00 ^c^	0.07 ± 0.01 ^b^
Benzaldehyde	0.17 ± 0.05 ^a^	0.00 ± 0.00 ^b^	0.00 ± 0.00 ^b^	0.01 ± 0.00 ^b^
Butanoic acid	0.37 ± 0.12 ^b^	0.59 ± 0.21 ^a^	0.00 ± 0.00 ^c^	0.00 ± 0.00 ^c^
Phenol	0.10 ± 0.03 ^a^	0.00 ± 0.00 ^b^	0.00 ± 0.00 ^b^	0.01 ± 0.00 ^b^
Trimethylpyrazine	0.15 ± 0.05	1.31 ± 0.47	ND	0.00 ± 0.00

ND: not detected. ^1^ All volatile compounds were identified by comparison with mass spectra and retention index database. Data are expressed as mean ± SD. Different letters are significantly different among groups (*p* < 0.05), ^NS^: not significant.

**Table 6:** Changes in pH, total acidity, and salinity of kimchi prepared with *jeotgal* alternatives.

Week	Samples	pH	Total Acidity (%)	Salinity (%)
0	CON	4.90 ± 0.00 ^a^	0.50 ± 0.01 ^c^	1.87 ± 0.04 ^a^
	JA1	4.54 ± 0.02 ^c^	0.79 ± 0.00 ^a^	1.13 ± 0.03 ^b^
	JA2	4.94 ± 0.01 ^b^	0.69 ± 0.00 ^b^	1.03 ± 0.02 ^b^
1	CON	4.35 ± 0.01 ^c^	0.73 ± 0.01 ^c^	1.85 ± 0.00 ^a^
	JA1	4.41 ± 0.01 ^b^	0.85 ± 0.01 ^b^	1.13 ± 0.01 ^b^
	JA2	4.55 ± 0.02 ^a^	0.97 ± 0.01 ^a^	1.03 ± 0.02 ^b^
2	CON	4.03 ± 0.01 ^c^	1.12 ± 0.02 ^b^	1.85 ± 0.01 ^a^
	JA1	4.23 ± 0.01 ^b^	1.15 ± 0.01 ^b^	1.12 ± 0.00 ^b^
	JA2	4.31 ± 0.02 ^a^	1.36 ± 0.02 ^a^	1.02 ± 0.02 ^b^

Data are expressed as mean ± SD. Different letters are significantly different among groups (*p* < 0.05).

**Table 7:** Microbial changes of kimchi prepared with *jeotgal* alternatives.

Week	Samples	Total Viable Bacteria (CFU/mL)	Lactic Acid Bacteria (CFU/mL)
0	CON	6.93 ± 0.03 ^b^	6.87 ± 0.02 ^NS^
	JA1	7.02 ± 0.01 ^a^	6.88 ± 0.05
	JA2	6.98 ± 0.03 ^b^	6.84 ± 0.02
1	CON	8.19 ± 0.06 ^a^	8.00 ± 0.05 ^NS^
	JA1	7.93 ± 0.05 ^b^	7.80 ± 0.07
	JA2	7.99 ± 0.04 ^b^	7.91 ± 0.01
2	CON	8.49 ± 0.01 ^b^	8.50 ± 0.05 ^b^
	JA1	8.83 ± 0.05 ^a^	8.74 ± 0.05 ^a^
	JA2	8.51 ± 0.12 ^b^	8.42 ± 0.04 ^b^

Data are expressed as mean ± SD. Different letters are significantly different among groups (*p* < 0.05), ^NS^: not significant.

**Table 8:** Free amino acid composition of kimchi prepared with *jeotgal* alternatives.

Free Amino Acids	Week	CON (mg/kg)	JA1 (mg/kg)	JA2 (mg/kg)
Glutamic acid	0	413.25 ± 2.19 ^c^	1246.23 ± 6.95 ^a^	686.59 ± 1.67 ^b^
	1	428.60 ± 3.65 ^c^	1057.81 ± 1.38 ^a^	796.84 ± 6.81 ^b^
	2	466.21 ± 2.88 ^c^	1231.37 ± 10.95 ^a^	910.89 ± 18.50 ^b^
Glycine	0	106.31 ± 0.83 ^a^	55.63 ± 0.45 ^b^	95.48 ± 0.03 ^a^
	1	114.99 ± 1.65 ^b^	59.46 ± 0.21 ^c^	124.98 ± 1.82 ^a^
	2	125.06 ± 0.30 ^b^	63.12 ± 0.25 ^c^	145.24 ± 2.88 ^a^
Arginine	0	429.60 ± 3.92 ^b^	467.05 ± 1.70 ^a^	446.23 ± 0.86 ^b^
	1	443.33 ± 6.29 ^b^	462.16 ± 2.55 ^b^	514.60 ± 4.76 ^a^
	2	460.33 ± 4.11 ^b^	487.34 ± 9.22 ^b^	569.39 ± 12.09 ^a^
Alanine	0	513.44 ± 3.09 ^b^	412.26 ± 1.32 ^c^	533.65 ± 2.09 ^a^
	1	526.47 ± 7.12 ^b^	408.20 ± 1.89 ^c^	718.40 ± 7.18 ^a^
	2	572.12 ± 4.73 ^b^	428.67 ± 4.48 ^c^	783.50 ± 16.69 ^a^
Taurine	0	46.13 ± 1.11 ^b^	26.50 ± 0.12 ^b^	293.33 ± 1.84 ^a^
	1	44.96 ± 1.03 ^b^	23.96 ± 0.54 ^b^	313.50 ± 2.68 ^a^
	2	44.70 ± 0.66 ^b^	25.11 ± 0.18 ^b^	341.98 ± 5.40 ^a^
GABA	0	243.81 ± 4.16 ^c^	477.81 ± 1.06 ^a^	342.11 ± 1.88 ^b^
	1	230.97 ± 3.67 ^c^	433.86 ± 1.94 ^a^	363.28 ± 4.60 ^b^
	2	224.44 ± 2.64 ^c^	463.39 ± 2.54 ^a^	352.96 ± 6.16 ^b^
Leucine	0	133.04 ± 1.17 ^a^	75.18 ± 1.14 ^c^	98.13 ± 0.13 ^b^
	1	139.06 ± 1.77 ^a^	81.77 ± 0.28 ^c^	124.31 ± 1.66 ^b^
	2	141.77 ± 1.52 ^a^	79.97 ± 0.92 ^c^	129.32 ± 3.03 ^b^
Isoleucine	0	169.45 ± 0.87 ^a^	83.12 ± 1.01 ^c^	142.35 ± 0.83 ^b^
	1	194.37 ± 1.75 ^a^	102.10 ± 0.17 ^b^	192.46 ± 3.33 ^a^
	2	218.71 ± 1.61 ^a^	113.11 ± 1.00 ^b^	228.27 ± 4.46 ^a^

Data are expressed as mean ± SD. Different letters are significantly different among groups (*p* < 0.05).

**Table 9:** Volatile compounds composition in kimchi prepared with *jeotgal* alternatives.

Volatile Compounds ^1^	Week	CON (Peak Area)	JA1 (Peak Area)	JA2 (Peak Area)
(2-Isothiocyanatoethyl) benzene	0	2.12 ± 0.20 ^a^	1.84 ± 0.08 ^b^	2.18 ± 0.11 ^a^
	1	0.73 ± 0.05 ^b^	0.91 ± 0.04 ^a^	0.64 ± 0.05 ^b^
	2	0.26 ± 0.06 ^b^	0.26 ± 0.00 ^b^	0.36 ± 0.02 ^a^
2,3-Butanediol	0	0.06 ± 0.01 ^NS^	0.07 ± 0.03	0.07 ± 0.01
	1	0.16 ± 0.03 ^b^	0.17 ± 0.00 ^b^	0.32 ± 0.07 ^a^
	2	1.09 ± 0.13 ^c^	1.60 ± 0.10 ^b^	2.39 ± 0.69 ^a^
3,7-Dimethyl-2,6-octadien-1-ol	0	1.67 ± 0.21 ^a^	1.13 ± 0.07 ^b^	1.01 ± 0.09 ^b^
	1	0.50 ± 0.05 ^a^	0.59 ± 0.02 ^a^	0.39 ± 0.03 ^b^
	2	0.21 ± 0.02 ^b^	0.19 ± 0.02	0.25 ± 0.03
4-Isothiocyanato-1-butene	0	2.50 ± 0.38 ^b^	2.96 ± 0.19 ^a^	2.44 ± 0.05 ^b^
	1	0.57 ± 0.01 ^b^	0.78 ± 0.00 ^a^	0.48 ± 0.00 ^c^
	2	0.05 ± 0.01 ^NS^	0.04 ± 0.00	0.06 ± 0.00
Acetic acid	0	0.12 ± 0.02 ^NS^	0.13 ± 0.01	0.09 ± 0.01
	1	0.51 ± 0.04 ^a^	0.34 ± 0.02 ^c^	0.42 ± 0.03 ^b^
	2	1.63 ± 0.16 ^a^	1.44 ± 0.06 ^b^	1.78 ± 0.02 ^a^
Benzenepropanenitrile	0	0.75 ± 0.13 ^NS^	0.86 ± 0.08	0.88 ± 0.02
	1	1.01 ± 0.04 ^a^	0.91 ± 0.04 ^a^	0.62 ± 0.02 ^b^
	2	1.04 ± 0.08 ^a^	0.76 ± 0.06 ^b^	0.77 ± 0.06 ^b^
Diallyl_disulphide	0	1.50 ± 0.18 ^NS^	1.44 ± 0.09	1.44 ± 0.09
	1	3.95 ± 0.28 ^a^	2.82 ± 0.16 ^b^	2.63 ± 0.15 ^c^
	2	3.68 ± 0.50 ^a^	3.17 ± 0.25 ^c^	3.48 ± 0.01 ^b^
Methyl-2-propenyl disulfide	0	0.95 ± 0.16 ^NS^	0.84 ± 0.04	0.97 ± 0.05
	1	2.13 ± 0.07 ^a^	1.69 ± 0.03 ^b^	1.33 ± 0.04 ^c^
	2	1.20 ± 0.15 ^a^	0.76 ± 0.09 ^b^	0.82 ± 0.00 ^b^
Methyl-2-propenyl trisulfide	0	1.78 ± 0.22 ^NS^	1.46 ± 0.12	1.86 ± 0.01
	1	1.26 ± 0.10 ^a^	0.96 ± 0.04 ^b^	0.98 ± 0.05 ^b^
	2	1.07 ± 0.15 ^b^	1.01 ± 0.08 ^b^	1.16 ± 0.04 ^a^
Zingiberene	0	1.63 ± 0.00 ^a^	1.64 ± 0.14 ^a^	1.18 ± 0.27 ^b^
	1	1.36 ± 0.20 ^a^	0.72 ± 0.15 ^b^	0.62 ± 0.06 ^b^
	2	1.45 ± 0.27 ^a^	0.74 ± 0.01 ^c^	1.14 ± 0.00 ^b^

^1^ All volatile compounds were identified by comparison with mass spectra and retention index database. Data are expressed as mean ± SD. Different letters are significantly different among groups (*p* < 0.05), ^NS^: not significant.

## Data Availability

The data presented in this study are available in the article.
